# Different Aspects of the Voltammetric Detection of Vitamins: A Review

**DOI:** 10.3390/bios13060651

**Published:** 2023-06-14

**Authors:** Denise Kiamiloglou, Stella Girousi

**Affiliations:** Analytical Chemistry Laboratory, School of Chemistry, Faculty of Sciences, Aristotle University of Thessaloniki, 54124 Thessaloniki, Greece; ntenizkl@chem.auth.gr

**Keywords:** vitamins, electroanalysis, nanomaterials, modified electrodes

## Abstract

Vitamins comprise a group of organic chemical compounds that contribute significantly to the normal functioning of living organisms. Although they are biosynthesized in living organisms, some are also obtained from the diet to meet the needs of organisms, which is why they are characterized as essential chemical compounds. The lack, or low concentrations, of vitamins in the human body causes the development of metabolic dysfunctions, and for this reason their daily intake with food or as supplements, as well as the control of their levels, are necessary. The determination of vitamins is mainly accomplished by using analytical methods, such as chromatographic, spectroscopic, and spectrometric methods, while studies are carried out to develop new and faster methodologies and techniques for their analysis such as electroanalytical methods, the most common of which are voltammetry methods. In this work, a study is reported that was carried out on the determination of vitamins using both electroanalytical techniques, the common significant of which is the voltammetry technique that has been developed in recent years. Specifically, the present review presents a detailed bibliographic survey including, but not limited to, both electrode surfaces that have been modified with nanomaterials and serve as (bio)sensors as well as electrochemical detectors applied in the determination of vitamins.

## 1. Introduction

Vitamins are low-molecular-weight organic compounds that play quite a substantial role in many metabolic pathways, contributing to the normal functioning of the body (e.g., in maintenance, development, reproduction) even in very small quantities. They also have the ability to prevent the manifestation of dysfunctions or ailments, which can occur when they are present in a small amount or not at all. They are a natural component of foods, in which they are usually found in a small concentration. As most vitamins cannot be synthesized by the body in sufficient quantities, their intake from food is necessary to meet the body’s physiological needs.

Almost all vitamins consist of a family of chemically related substances, called vitamins, and have the same or similar biological functions. They participate in basic functions of metabolism, functioning as: (1) coenzymes, (2) donors/recipients of H^+^/e^−^, (3) antioxidants, (4) hormones, and (5) effectors of gene transcription [1].

## 2. The Vitamins

The term “vitamin” was coined by biochemist Casimir Funk in 1912 [2], and was derived from the combination of the English words “vital” and “amine”, as Funk’s studies on organic food agents had showed that they were essential for life and had the ability to prevent nutritional diseases (e.g., beriberi, pellagra, scurvy) [3].

The nomenclature of vitamins is quite complex, and comprises combinations of the first studies that discovered them and the attempts of the International Association of Nutritionists in 1978 to create a standardized vocabulary that included all vitamins. According to the IUPAC (International Union of Pure and Applied Chemistry), the IUB (International Union of Biochemistry), and the ASN (Committee on Nomenclature of the American Society for Nutrition), the name of each vitamin essentially describes all the vitamins that constitute this particular family of vitamins [1]. Later, in 1916, each vitamin was defined with a specific capital letter in the Latin script.

Vitamins are classified by their solubility into the fat-soluble and water-soluble categories (Table 1) [1]. Water-soluble vitamins have one or more polar or ionizing groups (e.g., carboxyl-, keto-, hydroxyl-groups, amino acids, or phosphate groups), dissolve in polar solvents, and are synthesized by different metabolic pathways. In contrast, fat-soluble vitamins have mainly aromatic rings and aliphatic chains, dissolve in non-polar solvents, and have some common structural features.

Vitamins are widely used as additives in dietary supplements and pharmaceuticals, making their stability much more important. Fat-soluble vitamins and water-soluble vitamins C, B1, B2, and B7 have low stability to oxidation, and so it is necessary to protect them from oxygen, heat, metal ions (especially Fe^2+^ and Cu^2+^), polyunsaturated fatty acids, and ultraviolet light. For this reason, antioxidant substances are quite often used in the formation of these vitamins. When it comes to vitamins A and E, it is preferred to use their esterified forms due to their high stability. In contrast, vitamins B3, B5, B6, B9, and B12 are more stable.

The chemical and physical properties of vitamins affect their absorption. In the aqueous environment of the digestive system, fat-soluble vitamins form micelles with other present lipid molecules or those derived from dietary fats [4,5]. On the other hand, water-soluble vitamins, which are soluble in the polar environment of the intestinal lumen, can be absorbed directly from the surface of the intestine [1].

The postabsorptive transport mechanisms of vitamins also depend on their specific physical and chemical properties. A critical factor in the transport of vitamins from the small intestine to the liver and other organs is the degree of solubility of the vitamins in the aqueous environment of blood plasma and lymph.

The storage and distribution of vitamins in different tissues also depend on their chemical and physical properties. Generally, fat-soluble vitamins are contained and stored in combination with tissue lipids. For this reason, lipid-rich tissues (e.g., fat tissue and liver) often have large reserves of fat-soluble vitamins, which help animals receive small concentrations of vitamins at times when their dietary intakes are low.

### 2.1. Characterization of the Electrochemical Behavior of Vitamins

As is well known, vitamins are contained in dietary supplements and pharmaceuticals even in very small quantities. The effective determination of vitamins has been done by various analytical techniques, the main of which are chromatographic techniques (HPLC, Ionic chromatography, supercritical fluid chromatography coupled/not coupled with mass spectrometry, etc.), spectroscopic techniques (fluorescence, UV spectroscopy), immunochemical methods, chemiluminescence, chromatography-mass spectrometry (GC-MS), gas chromatography, and many others [6,7,8,9,10,11,12,13,14,15,16,17,18,19,20,21,22,23,24,25,26,27,28,29,30,31,32,33,34,35,36,37,38,39,40,41,42,43,44,45]. Although these techniques have applications in many fields, they have complicated sample processing, are time consuming, and require expensive equipment. For these reasons, the research interest has turned to electroanalytical techniques—mainly to voltammetric techniques—due to their advantages (low cost, fast results, high sensitivity, non-destruction of samples). Several voltammetric studies have also been reported where simultaneous analyses of two or three vitamins were performed. Water-soluble vitamins have been studied primarily using differential pulse voltammetry, square-wave pulse voltammetry, cyclic voltammetry, linear sweep voltammetry, and redistributive voltammetry. What should always be considered is that because these vitamins are in different concentrations and are on different substrates or means, the experimental conditions have to be adapted to the data [45]. The mechanisms carried out on a working electrode differ depending on the vitamin being examined. Simple electron transfer (as in B2, B3, B7, C, and D), simultaneous oxidation and reduction (as in B1, B5, B6, B9, and E), or two successive reductions (as in K and B12) can be made. On the other hand, vitamin A has complex electrochemical properties. The electrodes that have been used to determine vitamins are mainly metal electrodes Au, Pt, and Hg, hanging mercury drop electrode (HMDE), dropping mercury electrode (DME), carbon electrodes, glassy carbon electrodes (GCEs), carbon paste electrodes (CPEs), and diamond electrodes [45,46,47,48,49,50]. These electrodes have also been modified using more durable and efficient materials such as graphene, gold nanoparticles, or carbon nanotubes (CNTs) in order to increase their stability, functionality, and efficiency. In recent years, nano sensors, which are quite selective and provide results with low detection limits and high sensitivity, have also begun to be used as formatters. Of these, Hg electrodes have been used the most due to their advantages. However, due to the toxicity of Hg, a search has been conducted for alternative electrodes. Such alternative electrodes could use Hg either in amalgam form or in very small amounts, making it less dangerous. Alternative working electrodes based on solid amalgams (the glassy solid amalgam electrodes of type p-SAE or, after forming their surface by meniscus Hg, m-SAE) have also been developed [51,52]. The use of amalgam does not change these electrodes’ properties for some minutes, while their generated potential is comparable to that of the typical Hg electrode. Screen-printed electrodes (SPE) have also been used due to their low cost, ease of use, and high repeatability, as well as the lack of need to clean their surface.

#### 2.1.1. Fat-Soluble Vitamins

##### Voltammetric Properties of Vitamin A (Retinol)

Vitamin A is converted into its different forms through redox reactions, and this is why it can be studied by using electrochemical methods, mainly by using cyclic voltammetry. In other studies, the oxidation of retinol has been studied by using cyclic voltammetry with a glassy carbon electrode in a 0.05-M acetate buffer containing 95% methanol [53], with tetrahydrofuran (THF) containing tetrabutylammonium perchlorate [54], or with CH_3_CN/H_2_O containing LiClO_4_, at a potential equal to +0.8 V [54]. The mechanism of oxidation describes a chemically irreversible transfer of one electron per molecule, as only one oxidation peak appears in the voltammogram of the reaction. The oxidation of retinaldehyde in THF with tert-butyl to ammonium perchlorate (TBAP) occurs with a potential equal to +1 V vs. Ag/AgCl electrode (saturated with KCl) and results in retinol and other products [54]. The reaction is chemically nonreversible and occurs by transfer of up to four electrons per molecule. Retinaldehyde is oxidized to a glassy carbon electrode with a potential equal to +0.68 V according to the Fc|Fc^+^ standard electrode [54]. Many studies with cyclic voltammetry and square-wave pulse voltammetry have shown that retinoids can undergo reduction to Pt electrodes or to Pb^2+^ electrodes coated with Hg. Reduction reactions occur through the transfer of one electron in seconds (in cyclic voltammetry) but more electrons in minutes (in electrolysis) [55]. Studies have shown that carotenoids are oxidized in two single-electron transfer processes in a CH_2_Cl_2_ solvent, where they have good solubility, with a potential of between +0.5–+1 V according to an SCE electrode [56,57]. Additionally, many carotenoids can also be traced back to many single-electron processes in CH_2_Cl_2_ with potentials more negative than −1 V according to SCE. However, more attention has been paid to the oxidation mechanism because the important biological properties of compounds (i.e., antioxidant functions and photoprotection reactions) are realized through the cations. For the most carotenoids, the first oxidation reaction (E1) is performed with potentials +0.50–+0.72 V to SCE while the second oxidation (E2) varies between +0.52–+0.95 V to SCE [57]. In the case of β-carotene, only a chemically reversible oxidation of two electrons to β-carotene^2+^ (via formation of intermediate the β-carotene^+^) is observed [58]. The oxidation of β-carotene at a Pt electrode and in aprotic solvent CH_2_Cl_2_ has also been studied by using cyclic voltammetry. In one study, the mechanism showed a chemically reversible transfer of two electrons with simultaneous oxidation of β-carotene and cationic radical [55]. Oxidation reactions were performed with −2.3 V potentials vs. Fc|Fc^+^ electrode. The study of redox reactions at a hanging Hg drop electrode in DMF:benzene (2:1) has shown up to four separate reduction waves with peaks occurring at potentials of −1.68 V, −1.85 V, −2.49 V and −2.83 V according to SCE [59]. The different values of potentials are related to the different contents of water in the solvents CH_2_Cl_2_ and DMF.

Redox reactions of retinol and carotenoids have also been studied at a glassy carbon electrode in means containing surfactants. These substances can be used as less dangerous substitutes for organic solvents as they facilitate the adsorption and solubility of various electrochemically active substances [60].

##### Voltammetric Properties of Vitamin D

A few electrochemical methods have been applied to determine vitamin D3. These methods use bare or modified glassy carbon electrode (GCE) or Pt electrode as the working electrode [61,62,63,64]. A boron-doped diamond electrode (BDDE) has recently been used to study the electrochemical oxidation of cholecalciferol to determine the metabolites of vitamin D [62]. This electrode, in addition to its low cost, has shown several advantages in electrochemical analyses [65]. In one study, the non-modified BDDE electrode in selected supporting electrolytes that were prepared in 50% ethanol was used to determine vitamin D3 by using square-wave pulse voltammetry. The optimal oxidation peak was observed at a potential of +1.00 V and at an optimal pH of 5.0 [62]. The study of the forms D2 and D3 by using cyclic voltammetry showed that electron transfer takes place, followed by a rapid, non-reversible chemical step [55,61,66,67]. A study of vitamins D2 and D3 by using cyclic voltammetry with a glassy carbon electrode in methanol-containing acetate buffer showed that the oxidation of both compounds is a chemically irreversible process, since only an oxidation peak appeared on the voltammogram in the study. However, the number of electrons involved in the oxidation reaction is unknown [66]. The maximum oxidation potential (Ipox) in the study was found to be +1.1 V according to SCE, and no reduction reactions to negative potentials took place. This study, and another one done in ethanol [54], showed that vitamins D2 and D3 adsorb to the surface of the electrode during oxidation, thus polluting the electrode and reducing the generated current in subsequent scans [55,61]. It has been shown that the chemical oxidation with permanganate in ethanol forms a diol through a reaction taking place in the triene, and thus it is assumed that the electrochemical oxidation also takes place in triene [54,66].

Additional cyclic voltammetry experiments with the Ag|AgCl standard electrode on ethanol and supporting electrolyte (LiClO_4_) [55,61,66] showed oxidation with a potential of +1.08 V vs. to Fc|Fc^+^ electrode, which has also been confirmed by using cyclic dichroism experiments (CD) [55]. In studies involving organic solvents such as CH_3_CN and CH_2_Cl_2_, the typical potential was found at +1.4–1.5 V according to normal hydrogen electrode (NHE) [61]. The mechanism of oxidation shows that this oxidation, too, took place in the triene [55,61,66].

##### Voltammetric Properties of Vitamin E (α-Tocopherol)

Vitamin E has been studied quite a lot with voltammetry. It has been found that the intermediates that result from redox reactions of α-tocopherol have longer shelf lives than oxidative intermediates of other phenols [68,69,70,71]. The mechanism of redox reactions of α-TOH (also applicable to β-, γ- and δ-tocopherols) in organic solvents CH_3_CN or CH_2_Cl_2,_ which has been studied by using cyclic voltammetry [54], is done by a −2e^−^/−H^+^ transfer process. The exact mechanism of oxidation varies, depending on whether dissolved acids or bases are present in the organic solvents of the reaction. The absence of water drastically improves the stability of oxidized forms of vitamin E [71]. In contrast, the R (phytyl) group of α-TOH does not affect its electrochemical properties [54,55,72].

At a Pt electrode in CH_3_CN with supporting electrolyte 0.2Μ Bu_4_NPF_6_ and in the absence of an acid or base, α-TOH is oxidized by the transfer of an electron with a potential of +0.5 V versus Fc/Fc^+^ electrode to form the cationic radical α-TOH^+^, which is rapidly deprotonated to form the neutral radical, α-TO. Since the oxidation potential of α-TO is less than that of α-TOH, α-TO is further oxidized at the electrode surface to form the diamagnetic cation α-TO^+^ [54,55,69]. Overall, the oxidation mechanism can be considered to occur via electron transfer followed by a chemical step [54,70,73]. However, there is a possibility that the second electron transfer step takes place via deprotonation towards the formation of α-TO+ [54,71,74]. The mechanism of oxidation to CH_3_CN or CH_2_Cl_2_ is fully chemically reversible in milliseconds (in cyclic voltammetry) and in hours (in electrolysis), resulting in the application of a reducing potential that allows the original molecule to be quantitatively regenerated [72,73]. The first and reverse process are effected by the transfer of two electrons and one proton. The individual electron transfer stages occur at different potentials and are spaced apart for this, and there is a large separation at the potentials. The diamagnetic cations of β-, γ-, and δ-tocopherols have shorter lifespans than α-TO+. The long lifespan of α-TO^+^ has led to the conclusion that α-TO^+^ has an important biological role as an antioxidant in cell signaling [54,69]. The half-life of α-TO^+^ is largely determined by the moisture content of the solvent. In aprotic solvents, α-TO^+^ reacts with traces of H_2_O to form hemiacetal (α-TOQ(OH)), which then undergoes rearrangement, forming para-quinone [α-TOQ] [70,75,76]. Hemiacetal can be detected voltammetrically with a reduction peak with a potential of −0.3 V at a standard Fc/Fc+ electrode, while dicotone is detected with a reduction peak and a potential of −1.1 V at a standard Fc/Fc^+^ electrode at CH_3_CN [69,76]. However, at a low concentration of water (<0.5 M), the yield of para-quinone is low, with many oxidation products being produced that have not been characterized [76]. On the contrary, when oxidation of a-TOH takes place in solutions containing high percentages of water (such as ethanol mixtures), para-quinone is produced in high yield [77,78,79].

The oxidation of a-TOH in CH_3_CN or CH_2_Cl_2_ in the presence of dry acids or bases is done by a different mechanism than in pure solvents (i.e., those containing supporting electrolytes). In the presence of a strong acid (e.g., CF_3_SO_3_H or CF_3_COOH) the initial oxidation occurs by transfer of an electron to form the cationic radical α-TOH^+^. The α-TOH^+^ is further oxidized by transferring an electron with a potential of +1.4 V to a standard Fc/Fc^+^ electrode to form the α-TOH^2+^, which is immediately deprotonated (even in a strong acidic environment) to form the diamagnetic cation (α-TO^+^). In acidic conditions the oxidation of the α-TOH.+ occurs through the simultaneous transfer of electrons and protons to form the diamagnetic cation (α-TO^+^) [54,73]. In the presence of a strong dry lipophilic base, such as Et_4_NOH, α-TOH is immediately deprotonated to form the phenolic anion α-TO^−^ [68]. The neutral radical has a short half-life and either decays through a dimerization reaction to form a stereodimer or undergoes further oxidation by transferring an electron to the electrode surface to form α-TO^+^ [54]. Recent studies have shown that α-TO+ can be produced by the oxidation of a-TOH at the interface of two immiscible electrolyte solutions (ITIES) [80], and is likely to be produced in a standard biological laminate film deposited on the surface of the electrode [75].

##### Voltammetric Properties of Vitamin K

Vitamin K has been studied with square-wave pulse voltammetry in aprotic organic solvents that have small percentages of moisture. Results have shown that vitamin K, which has a quinone structure, undergoes reduction through two single-electron transport processes [54,55].

Because vitamin K’s electrochemistry exhibits a strong dependence on the water content of the solvent, in vivo typical potentials are highly dependent on the concentration of protons in the solvent, as each oxidative state of K has different pKa values. For example, in the aprotic solvent CH_3_CN, vitamin K1 is first reduced by transfer of an electron with a potential of −1.2 V versus the standard Fc/Fc^+^ electrode forming the radical anion K1^−^ (hemicinone radical) (Equation (1)).

K1 + e^−^ ⇄ K1^−^ E^0^_f(1)_/V (1)

The radical K1^−^ is then reduced by losing an electron with a potential of −1.7 V versus the Fc/Fc+ electrode forming the dianon K_1_^2−^ (Equation (2)) [81].

K1^−^ + e^−^ ⇄ K1^2−^ E^0^_f(2)_/V (2)

In both steps, the transfer of electrons is chemically reversible, resulting in the two vertices being of the same size the voltammogram. The reaction is also chemically and electrochemically reversible in solvents DMSO, butylnitrile (C_3_H_7_CN), CH_3_CN, and dichloromethane (DCM). The first step of the reaction has a different potential from that of molecular oxygen reduction, and so it is important to remove the dissolved oxygen of the solution when applying voltammetric methods to determine K1 [54,81]. The determination of reduction potentials (E^0^_f(1)_ and E^0^_f(2)_) in Equations (1) and (2) is difficult because the potentials are affected by the moisture of the solvent [54,81]. The reduction potentials of many quinones, for both single-electron processes, shift to more positive potentials as more water is added to the solvent. This effect is due to strong hydrogen bonds between the dianion and water [54,81]. The more water added to the solvent, the more positively the second reduction reaction shifts. If too much water is added to the solvent, the two single-electron processes merge into a two-electron process, as has been observed in voltammetric experiments of K1 in a CH_3_CN solvent when the water concentration approached 7 M with a potential of −1.1 V versus the Fc|Fc^+^ electrode [54,55,81]. Although moisture is always present in organic solvents, the concentration of water is very rarely taken into account in measurements [82]. Thus, recent studies have used the voltammetric behavior of K1 in the presence of water to determine the content of water contained in solvents [54,81]. The determination of vitamin K by using voltammetry has also been performed in an aqueous environment over a pH range, either by depositing it as an oil on an electrode surface, binding it to an electrode surface by using sulfur binding, or by incorporating it into the phospholipid or alkanothiol layers located on the electrode surface and placing the electrode in an aqueous solution [54]. In low-pH aqueous solution, vitamin K1 undergoes reduction by two e^−^ and two H^+^ (+2e^−^/+2H^+^) to form hydroquinone (K1H_2_) [54]. The reduction reaction is chemically reversible and takes place in one step, although it is possible that simultaneous e^−^ and H^+^ transfer reactions take place. In aqueous media, the reverse oxidation of hydroquinone occurs through −2e^−^/−2H^+^ processes and appears as a peak on the voltammogram. The reduction of K1 in a high pH aqueous environment forms the dianon (K1(H_2_O)_2_)^2−^ via hydrogen bonds and is done in one step [54].

#### 2.1.2. Water-Soluble Vitamins

##### Voltammetric Properties of Vitamin C (Ascorbic Acid)

Ascorbic acid has been quite studied due to its important biological role with spectrophotometry, HPLC chromatography, fluorescence, and chemiluminescence. Among the electroanalytical methods, potentiometry, polarography, and voltammetry (cyclic and differential pulse) are most the used [83,84,85]. Electroanalytic methods for studying ascorbic acid are based on its oxidation to dehydroascorbate via a transfer mechanism of two e^−^ and two H^+^ [86]. The pH of the reaction solution affects the reaction mechanism. If the pH is less than the pKa_1_ of ascorbic acid (pKa_1_ = 4.5), two-proton exchange takes place, while at higher pH values, one-electron exchange takes place with the ascorbate anion. Ascorbic acid and dehydroascorbic acid are not stable at alkaline pH. In contrast, the ascorbate anion is oxidized to diketolactone, which is then dehydrated to dehydroascorbic acid. Dehydroascorbic acid is then converted to diol, which is oxidized at large potential values. A study of the oxidation of ascorbic acid at Au electrodes has shown that it takes place in two stages, wherein the first two-proton exchange takes place in a pH range of 2–4.5 followed by the exchange of one proton as the pH increases from 4.5 to 8. At pH higher than 8, two-proton exchange occurs [87]. However, at a pH of less than 4, oxidation to dehydroascorbic acid takes place followed by a non-reversible hydration to the formation of an inert product (2,3-diketogulonic acid), which accumulates on the surface of the electrodes, resulting in their contamination and functionality reduction [87]. For this reason, molded electrodes are mainly used in voltammetric studies of ascorbic acid. Molded carbon paste electrodes with transition metal complexes have wide usage as they have the ability to catalyze oxidation or reduce the substance to be analyzed. The use of the formative material reduces the potential required for the oxidation reaction, increasing the sensitivity of the electrode. It has been found that Co^3+^ and Fe^3+^ complexes have the highest electrocatalytic activities for this and are used for the electrocatalytic determination of many organic and biologically important compounds.

The oxidation of ascorbic acid has been studied by using square-wave pulse voltammetry with a carbon paste electrode, molded with Fe(III)-Y zeolite in a phosphate buffer of 0.1 M and pH = 5, where ascorbic acid has great stability [88]. The results showed that the electrocatalytic oxidation of ascorbic acid on the surface of the formed electrode with Fe(III)-Y is controlled by diffusion and described by a two-step process involving the (1) oxidation of ascorbic acid by Fe^3+^ and (2) the subsequent reoxidation of the resulting Fe^2+^. These processes occur at the solution-electrode interface, where Fe^3+^ is released through the exchange of ions with Na^+^ [88].

##### Voltammetric Properties of Vitamin B1 (Thiamine)

The determination of thiamine with analytical methods should be done carefully, selectively, and with great sensitivity, due to the presence of possible interferences and the low concentrations of the determined substance. The electroanalytic study of thiamine has been done by using polarography, potentiometry, and voltammetry. Voltammetric study has been done primarily with stripping voltammetry, using dropping Hg electrode, carbon paste electrode, and glassy carbon electrode. Many of these methods are not selective, but they involve time-consuming and expensive procedures. A highly sensitive method involves stripping voltammetry to determine thiamine in traces. One study using this method was done on a NaHPO_4_/NaH_2_PO_4_ mixture of 0.01 mol/L at pH = 6.2. The deposition potential was determined by scanning voltammetry, and thiamine was found to exhibit strong adsorption at +1.4 V. This method is based on the adsorption of thiamine on the electrode surface and on the catalytic effects it carries out which result in the increase of the sensitivity of thiamine [89].

The oxidation reaction of vitamin B1 has been studied with voltammetry using molded carbon paste electrodes in alkaline conditions (pH = 10), and has been characterized as a two-electron process [55] (Equation (3)).

(C_12_H_17_N_4_OS)^+^ ⇄ (C_12_H_14_N_4_OS) + 3H^+^ + 2e^−^ (3)

The mechanism involves an electron transfer stage followed by a rapid chemical reaction. This can be attributed to the non-active protonated form of vitamin B1. The presence of two vertices in the voltammogram indicates that after the release of an electron, a stable intermediate product is formed. At higher potentials, a second electron is released, resulting in the formation of thiochromium. This was confirmed in one study by applying square-wave pulse voltammetry to the unformulated carbon paste electrode, where two distinct peaks were observed for vitamin B1 with approximately the same height at 400 and 600 mV (according to the standard Ag/AgCl electrode). The oxidation potential equaled ~0.4 V according to standard Ag|AgCl electrode. In glassy carbon electrodes, oxidation occurs with a potential of +0.330 V versus standard Ag|AgCl potential and does not change when the pH is increased from 9.0 to 13.0. Additionally, a study was done to examine the reduction of thiamine pyrophosphate by using cyclic voltammetry at a Pt electrode and in a 1 M KCL solution [48]. The mechanism in the study described a potentially reversible transfer of 4 electrons with a potential of −0.58 V versus the standard Ag|AgCl electrode, which was activated when the N of the pyrimidine ring was protonated. As pH increased, the cathodic and anodic peaks decreased, but their potentials did not shift; however, the reduction of thiamine pyrophosphate was independent of pH.

The oxidation of vitamin B1 has also been studied with non-molded electrodes; however, the researchers studying this noted that there was an inability to distinguish the peaks, oxidation took place at a slow rate, and there was a risk of poisoning the electrode surface. To avoid this poisoning, a study using cyclic voltammetry to examine the oxidation of vitamin B1 was performed on a carbon paste electrode molded with a metallophthalocyanin complex (MnPc). The results showed a large increase in the oxidation current of the first wave, which was indicative of the catalytic action of the molded CPE electrode with MnPc, even after twenty scans. The electrocatalytic action of MnPc usually involves the oxidation of the MPC complex followed by electron transfer [89]. Another important advantage of this method is the possibility of direct measurement of vitamin B1 without complex sample preparation or derivation.

##### Voltammetric Properties of Vitamin B2 (Riboflavin)

**Figure 1:**
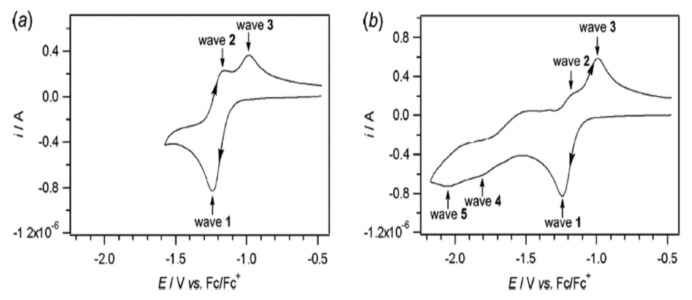
Cyclic voltammogram of reduction of 1 mM vitamin B2 at 1 mm diameter Pt electrode in DMSO with 0.5 M nBu_4_NPF_6_ at scan rate 0.100 V/ s (**a**) Scan range −1.6 V to −0.5 V vs. Fc/Fc+ (**b**) Scan range −2.2 V to −0.5 V vs. Fc/Fc+ [90].

The electrochemical behavior of vitamin B2 is complex, as has been noted in quinones [55]. The reduction of riboflavin involves two successive one electron steps lectron, resulting in the formation of an intermediate radical anion (flavosemicinone radical). The overall process depends significantly on the availability of protons. There are three oxidation states: the fully oxidized flavoquinone (Fl_ox_, vitamin B2), the flavosemicinone radical (Fl^−^), and the reduced two−electron flavohydroquinone (Fl^2−^_red_), each with three protonation states (Figure 1). The voltammetric study of vitamin B2 has been evaluated in water and aprotic solvents. In aprotic solvents lower degrees of protonation are common. The mechanism in aprotic solution is an electron transfer process followed by chemical steps, as shown in cyclic voltammograms recorded between −0.5 and −1.6 V versus the Fc|Fc^+^ electrode. In aqueous solution, B2 undergoes a reversible reduction process involving two electrons and two protons. The typical potential at normal pH is −0.21 V according to normal hydrogen electrode (NHE) [55].

##### Voltammetric Properties of Vitamin B3 (Niacin/Nicotinic acid)

The electrochemistry of nicotinic acid is complex due to its acid-base character. The dissociation constants pKa and pKb for nicotinic acid are 2.79 and 4.19, respectively. During the dissolution of nicotinic acid in water the amphoteric ion is formed by the deprotonation of carboxylic acid and protonation of pyridine. The various protonated forms of nicotinic acid shift typical potentials and make it difficult to interpret kinetic mechanisms. At normal pH the form of the amphoteric ion prevails [55]. The behavior of nicotinic acid is pH-dependent and has been studied with differential pulse voltammetry on a glassy carbon electrode (GCE) and a phosphate buffer. At the pH of the solution, the amphoteric ion prevails and one-electron transfer takes place [55].

The reduction of nicotinic acid has also been studied with cyclic voltammetry. The mechanism here describes electron transfer followed by a chemical reaction that consumes the reduction product and converts it into an inactive product. The reduction reaction is non-reversible and depends not only on the pH, but also on the type of the material with which the electrode was made. In one study, at an Hg electrode, with the degassing of the electrolyte and pH = 5.1, the reduction was made with a potential of −1.47 V with respect to an SCE electrode. At a hanging Hg drop electrode and at pH = 7.2 the reduction was completed with a potential of −1.5 V according to SCE. On a Pt electrode, nitrogen degassed, and with a KCL electrolyte the reduction did not yield a voltammetric signal in the range of potentials between +0.3–+0.8 V according to standard Ag|AgCl electrode. The most effective electrodes seem to be the diamond electrodes with admixtures of Hg and boron. In a boron-doped diamond electrode, reduction with potential −1 V to standard Ag|AgCl electrode was observed in one study, while reverse oxidation was found to take place at positive values. The negative value of the potential indicated that the solution was probably electrolyzed. When the concentration of protons increased, nicotinic acid was reduced more easily as the potential shifted to more positive potentials, resulting in a sharp increase in the size of the current.

##### Voltammetric Properties of Vitamin B5 (Pantothenic Acid)

A few electroanalytical studies have been reported for vitamin B5, and they have mainly focused on its active form, D-Panthenol. D-Panthenol is the alcoholic derivative of pantothenic acid and is used in electroanalytical studies because it is more stable in aqueous solutions [55]. The reduction study of panthenol has been done with cyclic voltammetry, differential-wave pulse voltammetry, and linear sweeping voltammetry, using carbon paste electrodes (CPE) molded with cobalt oxide catalysts in phosphate buffers at pH 6.08. The reduction mechanism here consists of a transfer of two electrons and two protons and is a non-reversible process controlled by diffusion. In linear sweeping voltammograms the reduction potential E_1/2_ is close to −0.4 V according to the standard Ag/AgCl electrode. A study of the oxidation of D-Panthenol was done with unformulated glassy carbon electrodes in a phosphate buffer for a specific pH range. The mechanism of oxidation was found to occur by transfer of two electrons and two protons. At pH values such that 7.0 ≤ pH ≤ 9.2, the peak potential varied linearly with pH [91].

##### Voltammetric Properties of Vitamin B6 (Pyridoxine)

**Figure 2:**
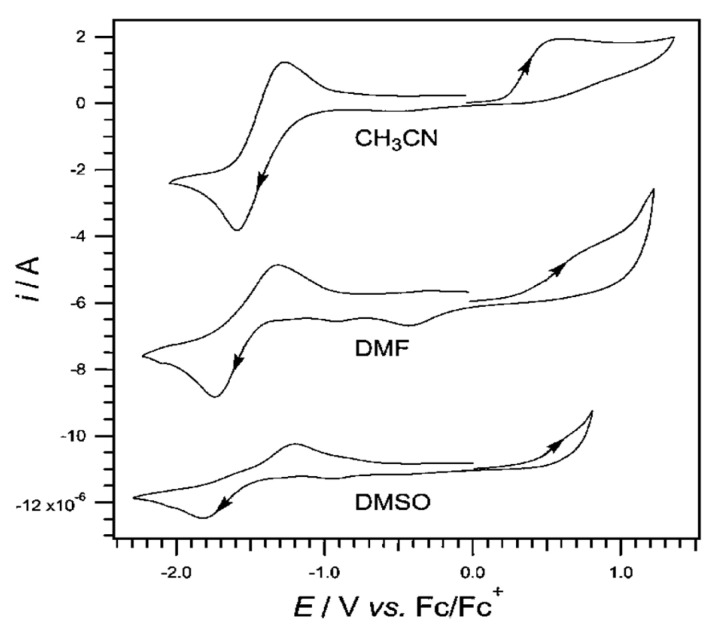
Cyclic voltammogram of 2 mM pyridoxine and nBu_4_NPF_6_ in CH_3_CN, DMF, and DMSO with a scan rate 0.1 V/s versus the Fc|Fc^+^ electrode [92].

Pyridoxine has been studied with cyclic voltammetry at Pt electrode in aprotic solvents CH_3_CN, DMF, and DMSO with the addition of nBu_4_NPF_6_ 0.2M (Figure 2) [55].

The oxidation of pyridoxine at CH_3_CN has been quite studied and is carried out in two stages. On the voltammogram, at the end of the first cycle, a second, small reduction wave with a peak of −0.7 V versus the standard Fc|Fc^+^ electrode is observed. At the start of the second stage, oxidation with a peak close to +0.6 V versus the standard Fc|Fc^+^ electrode is observed. In organic solvents, pyridoxine undergoes single-electron oxidation followed by a chemical step and then a second electron transfer step. The mechanism describes a chemical non-reversible oxidation with a potential of +0.5 V versus the standard Fc|Fc^+^ electrode.

The study of the electrocatalytic oxidation of pyridoxine has also been done using a glassy carbon electrode molded with dsDNA, which is immobilized on the electrode surface and oxidized with a potential of +0.58 V in alkaline solution [93]. The strong alkaline conditions of the solution denature dsDNA into ssDNA. According to the mechanism of the reaction, the oxidation of denatured ssDNA is a non-reversible process. The value of the potential increases as the concentration of DNA on the surface of the electrode increases, while correspondingly, it decreases as the concentration of DNA decreases. The mechanism describes electron transfer from guanine to DNA, which adsorbs to the surface of the glassy carbon electrode. This study showed that DNA can electrocatalytically oxidize vitamin B6 while providing greater protection of the nucleic bases on the electrode surface [94].

##### Voltammetric Properties of Vitamin B7 (Biotin)

Determination of vitamin B7 with voltammetry has been studied in the aprotic solvents DMF and DMSO. The reduction potential has been determined to be −1.6 V at DMF and −1.8 V at DMSO versus the Fc|Fc^+^ electrode. The reduction mechanism of biotin has been characterized as slow, non-reversible heterogeneous electron transport. The complete reduction of biotin occurs by one-electron transfer. In the scanning voltammogram, the separation of the anodic and cathodic peaks is very large and the potential arises as greater than +0.350 V [95]. In relevant studies conducted with glassy carbon electrodes in DMF and DMSO, no signals were recorded. In contrast, the catalytic reduction of biotin has been achieved at the Pt electrode [55]. Dihydrogen is formed either by the reduction of two biotin molecules or by the dimerization of adsorbed hydrogen atoms on the surface of Pt.

##### Voltammetric Properties of Vitamin B9 (Folic Acid)

**Figure 3:**
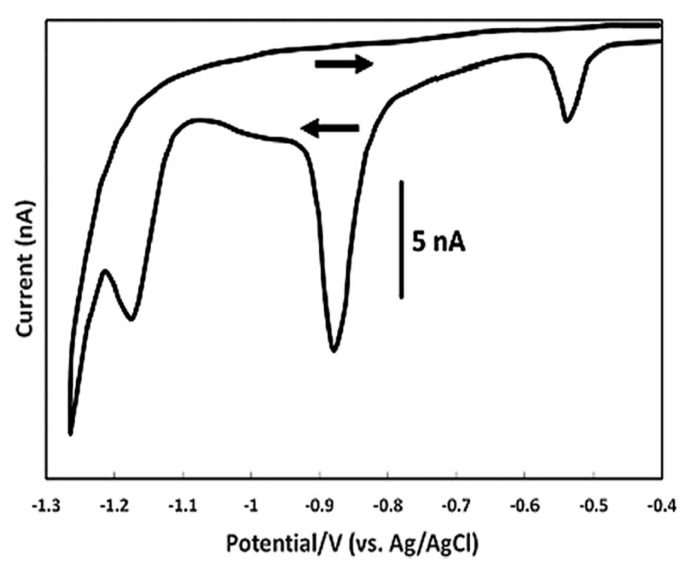
Cyclic voltammogram of the reduction of 100 nM folic acid at a hanging Hg drop electrode in acetate buffer solution pH = 5.2, at a scan rate of 0.020 V s^−1^ [97].

The electroanalytic study of folic acid with cyclic voltammetry has been done using several kinds of electrodes such as the dropping Hg electrode (Figure 3), the hanging Hg drop electrode, the glassy carbon electrode molded with polypyrrole phosphomolybdate film, the carbon paste electrode molded with p-tert-butylcalix(6)arene [CME-6], and the glassy carbon electrode molded with single wall carbon nanotubes (SWNTs) [96]. However, there is a disadvantage when using these electrodes, and this is the adsorption of folic acid on the surfaces of the electrodes through the benzene ring, resulting in their pollution. The study of folic acid reduction with dropping Hg electrode has given a potential of +0.71 V while the study of the oxidation with carbon paste electrodes, molded with p-tert-butylcalix(6)arene at pH = 5.0, has given a potential of +0.86 V versus the SCE electrode [96].

Folic acid’s reduction was also studied with an Au electrode molded with 2-mercaptobenzothiazole film (MBT/SAM) in a phosphate buffer solution of pH = 7.4. The potential was found to be +0.298 V and +0.116 V versus the SCE electrode, consistent with the results of other studies. The decrease of the potential in the reverse scanning indicates that a slower reaction follows [55]. The redox reaction of folic acid is highly dependent on pH as transfer of the conjugated protons takes place. In acidic means, all reduction reactions are chemically non-reversible and peak potentials shift to negatives as pH increases. Another study with a dropping Hg electrode, in aqueous solution NaClO_4_ at pH = 4, gave four reduction peaks with potentials of 0.045 V, −0.55 V, −0.85 V, and −1.0 V versus the standard Ag|AgCl electrode. At a dropping Hg electrode in acetate buffer (pH = 5.2) the reduction potentials were −0.5 V, −0.8 V and −1.2 V versus the standard Ag|AgCl electrode. The reduction mechanism that was proposed consisted of the following 4 steps, wherein each transfer is made by two electrons and two protons: (1) first there was a reduction of two electrons to 5,8-dihydrofolic acid with a potential equal −0.3 V versus the normal hydrogen electrode (NHE), (2) then a tautomerization to 7,8-dihydrofolic acid was performed, (3) a second reduction of two electrons to 7,8-dihydro-6–methylpterin was followed, (4) a third reduction of two electrons to 7,8-dihydro–6-methylpterin was performed, and (5) a third reduction of two electrons to 5,6,7,8-tetrahydro-6-methylpterine was performed [55].

Another study of folic acid’s oxidation by using cyclic voltammetry was done with an Au electrode, molded with a multi-walled Au/NPs carbon nanotube, in a phosphate buffer (pH = 2.5) which had a very low detection limit (1.0 × 10^−8^ mol/L). Such solid electrodes have good sensitivity to folic acid, but often have complex construction and preparation and high cost. The mechanism in the paper described a two-electron process, with an estimated potential equal to 0.79 V versus an SCE electrode, that was controlled by adsorption [98]. When the substrate was inert to the nanoparticles, the potential was shifted towards the negatives, and when the metal nanoparticles interacted with the substrate, the potential was positively shifted. The latter was affected by the possible formation of alloys and/or intermetallic compounds at the metal-substrate interface [99]. The adsorption of folic acid on the surface of the activated electrode caused a decrease in the generated current at the working electrode. An increase of the concentration of folic acid caused a further decrease in the peak current. This phenomenon was due to the presence of the amine group (–NH) of the folic acid ring that could adsorb to the surface of the gold [99].

##### Voltammetric Properties of Vitamin B12 (Cobalamin)

The three main oxidation states of vitamin B12 are Co^3+^, Co^2+^, and Co^+^, each of which has different chemical properties. The Co^3+^ ion is electronophilic, Co^2+^ acts as a radical, and Co^+^ is nucleophilic [100]. During the reduction of Co^2+^→Co^+^, the more powerful the axial substituent (H_2_O < OH− < CN− CH−) is, the more the transfer of electrons slows down, while the potential is negatively shifted. The thermodynamics and kinetic behavior of electron transport are influenced by the strength of the axial substituent. The density of the current caused by the flow of electrons and the pH influence the rate of redox reactions [55]. The reduction of B12 with axial substituent water molecules shows two one-electron reduction waves for the metal center (Co^3+^→Co^2+^→Co^+^). When the substituent is a strong base, a two-electron reduction wave [Co^3+^→Co^+^] is observed.

## 3. Vitamin Analysis Using Electroanalytical Techniques

Many studies of vitamin analysis have been carried out with electroanalytical techniques. This chapter describes the electroanalytical techniques of analyzing all vitamins in various samples with an emphasis on voltammetric techniques.

### 3.1. Fat-Soluble Vitamins

#### 3.1.1. Vitamin A

The determination of vitamin A in foods has been studied by using square-wave pulse voltammetry that uses carbon paste electrode (CPE) molded with Pt:C nanoalloy in ionic liquid (IL). This technique exhibits good sensitivity with a linear response between 0.1–100 M and with an LOD equal to 0.04 M [101]. The electrochemical determination of retinol has also been done by using differential pulse voltammetry with carbon paste electrode molded with sodium dodecyl sulfate surfactant (CPE/SDS). The method has turned out to be linear with low detection limits (up to 4.6 × 10^−7^ M) [102]. A simultaneous determination of retinol and Vitamin E in CH_3_CN has also been done with cyclic voltammetry with glassy carbon electrode (GCE) molded with the synthetic nanomaterial MWCNT [101].

#### 3.1.2. Vitamin D

**Figure 4:**
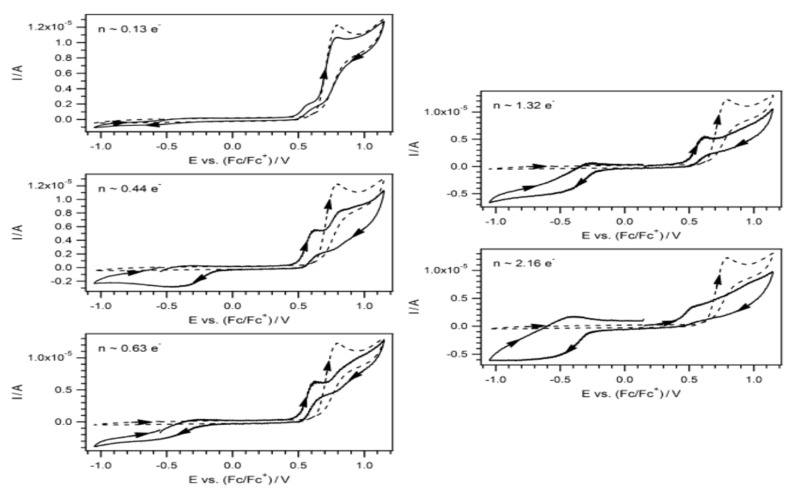
Cyclic voltammograms of vitamin D_3_ [61].

The determination of vitamin D2 has been done with a combination of cyclic voltammetry and differential pulse voltammetry using a glassy carbon electrode coated with a thin synthetic indium-titanium oxide (ITO; CD-CH) layer [103]. In order to determine vitamin D3, this electrode was combined with polyacrylonitrile nanofibers (PANnFs) and nanoparticles (NPs) of magnetite (Fe_3_O_4_) and studied with differential pulse voltammetry. PANnFs are very soluble and have thermal and mechanical stability, while magnetite nanoparticles are biocompatible and non-toxic [104]. A better determination of D3 was done with the glassy ITO electrode, molded with a gadolinium oxide sensor NRs (Gd_2_O_3_NRs), combined with aspartic acid (Asp-Gd_2_O_3_NRs) and with differential pulse voltammetry analysis [61]. In Figure 4 are shown cyclic voltammograms of vitamin D_3_.

Vitamin D3 has also been determined in pharmaceutical samples with a combination of cyclic voltammetry and differential pulse voltammetry using glassy carbon electrode molded with Ni(OH)_2_ particles in an inorganic SiO_2_/graphene oxide matrix. This method exhibited great selectivity and sensitivity, while the limit of detection was very low [105]. In analysis of blood samples, the detection of vitamin D was done with cyclic voltammetry using a glassy carbon electrode molded with an electrochemical sensor consisting of a synthetic film of bimetallic nanoparticles Cu-Ni and fullerene-C60. The method proved quite effective and showed good sensitivity [106]. Another technique for detecting vitamin D in human plasma samples has recently been developed by means of a reconstructive adsorbent voltammetry using a screen-printed carbon paste electrode (SPCE) coated with a polymer (MIP) combined with p-phenylenediamine resorcinol [107]. Another recent combination study of cyclic voltammetry and differential pulse voltammetry used a cerium (IV) oxide immunonanosensor (nCeO_2_/CC), which was prepared in carbon fabric (CC) by electrophoretic deposition of the nanomaterial (nCeO2). The method was applied to biological samples with good repeatability and high sensitivity [108].

#### 3.1.3. Vitamin E

Differential pulse voltammetry experiments on Pt microelectrodes to quantify vitamin E in solutions of vegetable oils and fats in N-methyl-2-pyrrolidine (NMP) solution with 0.2 M electrolyte nBu_4_NPF_6_ gave a potential of +1.04 V versus the standard Ag|AgCl electrode [55]. In another study, a combination of cyclic voltammetry and differential pulse voltammetry was applied to analyze simultaneously vitamins E and K in dietary supplements using Au electrodes molded with polyaniline (PAn) and γ-aluminium oxide (c-Al_2_O_3_), forming the nanomaterial PAn/c-Al_2_O_3_ [109]. On the other hand, to determine the active forms of E and K (quinones and tocopherols) in dietary supplements, adsorptive stripping square-wave voltammetry was applied to a glassy carbon electrode. The method had excellent sensitivity while being a low-cost and simple technique [110]. In another quantification study of α-, γ- and δ-tocopherols in oil samples, a square-wave pulse voltammetry was applied to a CF UMEinBz/ EtOH disk (1: 2) with 0.1 mol/L H_2_SO_4_ [111]. The results were processed using chemometric methods with two multivariate calibration models, MCR-ALS (multivariate curve resolution-alternating least squares) [112], and SandANNs (artificial neural networks (ANNs)) [113]. The advantages of these chemometric methods are their ability to obtain quantitative information from overlapping signals through mathematical models and the fact that no prior pretreatment of the sample is required. However, a disadvantage that can arise in the application of electroanalytical techniques is the lack of selectivity.

A recent study conducted a simultaneous identification of vitamins E and K in dietary supplements. The method involved a glassy carbon electrode in which an ex situ accumulation of the vitamins on the electrode surface was first performed. The vitamins were then detected by using adsorptive stripping square-wave voltammetry in a solution of 0.01 mol/L HNO_3_ containing 0.1 mol/L KCl at pH = 2.08. Both vitamins were detected in the anode, while the method turned out to be quite selective and highly sensitive [110].

#### 3.1.4. Vitamin K

For the study of the reduction of phylloquinone with cyclic voltammetry a glassy carbon electrode has been used on which a reversible adsorption reaction takes place [114]. In plant foods, vitamin K1 has been detected with a square wave adsorptive stripping voltammetry, preceded by the adsorption of the vitamin on the surface of the solid glassy carbon electrode. This method turned out to be quite sensitive and effective [115]. Additionally, the analysis of vitamin K2 in pharmaceutical preparations samples and foods has been done with cyclic voltammetry with a glassy carbon electrode, bare and modified [114,116]. The polyelectrode Pt, modified with a liquid amalgam film (RAgLAFm-E), has also been used for the quantification of K2. In recent years, many electrochemical sensors have also been developed to detect K, triggering the study of the manufacture of optical fiber sensors, which have a fairly low cost but very high sensitivity [117]. In blood plasma, vitamin K1 has been detected with cyclic voltammetry using pencil graphite electrode (PGE) modified with Ag nanoparticles and 2-amino-5-chlorobenzophenone (2-A-5-CBP), which were deposited by electro deposition on the surface of the PGE [118]. Additionally, for the determination of vitamin K3 in animal pharmaceuticals, a sensor consisting of a glassy carbon electrode molded with an imprinted poly(3,4-ethylenedioxythiophene) film (PEDOT) has been used [119]. The method has been applied with a combination of cyclic voltammetry and linear sweeping voltammetry, and has had very good sensitivity and detected K3 in traces. Additionally, an attempt has been made to simultaneously determine all four vitamins—A, D, E and K—by using square-wave adsorptive stripping voltammetry with a glassy carbon electrode. However, because all four vitamins are deposited on the surface of the electrode, the method has not shown selectivity and has not been able to separate them from each other. This is notably the case for vitamin D3. The simultaneous determination can be achieved by modifying the electrode with carbon nanomaterials [120].

### 3.2. Water-Soluble Vitamins

#### 3.2.1. Vitamin C

A recent study carried out the determination of vitamin C in fruit juices and creams with a combination of cyclic voltammetry and differential pulse voltammetry using a Hydroxyapatite-ZnO-Pd NPs modified carbon paste electrode (HAP-ZnO-Pd NPs/CPE) [121]. The voltammetric determination of ascorbic acid was also done using a disposable printed carbon electrode, bare and molded with a synthetic ZnO/Al_2_O_3_ nanosensor, which showed high selectivity, repeatability, and sensitivity. The anodic peak potential for the oxidation of ascorbic acid with the ZnO/Al_2_O_3_/SPE sensor is approximately 335 mV compared to 370 mV for the bare SPE. This shows that ZnO/Al_2_O_3_ nanomaterial improves the oxidation signal of ascorbic acid [83].

Differential pulse voltammetry with a graphene-molded carbon paste electrode has been successfully applied to determine ascorbic acid. Graphene greatly enhances the intensity of the current of a redox reaction as it is a good conductor. In this way the sensitivity of the method has been increased [122]. Differential pulse voltammetry has also been applied to pharmaceutical analysis using molded carbon paste electrodes with porphyrins. This combination has given low detection limits to the method (up to 1.1 × 10^−14^ mol/L) [123].

The successful determination of ascorbic acid with high sensitivity has also been done with cyclic voltammetry with a glassy carbon electrode molded with a polymeric film of poly [bromocresol purple], which enhances the signal of the generated current [124]. With cyclic voltammetry, a formed hyaline carbon electrode with a polymeric film (3-(5-chloro-2-hydroxyphenylazo)-4,5-dihydroxynaphthalene-2,7-disulfonic acid) has also been used in a solution with pH = 4, where a successful detection of ascorbic acid in a mixture of substances has been done [125]. The glassy carbon electrode has also been successfully modified with multi-wall carbon nanotubes and synthetic carbon nanotubes with [poly] xanthurenic acid. The formation with carbon nanotubes leads to great stability of the electrode at various pH values, low detection limits, and very good sensitivity [126,127]. Cyclic voltammetry with iodine-coated Pt electrode in 0.1 M KCL solution has been successfully applied for the selective determination of ascorbic acid in multivitamin pharmaceutical products (B1, B6, B12, B9, and C). The oxidation peak of ascorbic acid has occurred at 0.28 V, while the detection limits have been quite low [128]. Pharmaceutical formulations and foods have been studied with a combination of cyclic voltammetry and differential pulse voltammetry, with a carbon paste electrode molded with multi-walled carbon nanotubes and graphite in acidic solution with pH = 2 [129]. The successful determination of ascorbic acid in commercial samples has also been done with cyclic voltammetry, using bare carbon paste electrodes (PCPEs) and molded carbon paste electrodes (MCPEs) with calixarenes, such as p-tert-butylcalix(4)arene, in ammonium acetate (pH = 5) containing Pb^2+^ ions [130]. MCPEs have displayed better electrocatalytic activity. The greater catalytic action of MCPE can be explained by considering that the Pb^2+^ ion is strongly held in the p-tert-butylcalix(4)arene cavity, acting as an electron transport bridge, allowing it to interact with the ascorbic acid in the solution while oxidizing it. As the oxidation of ascorbic acid depends on pH, with its increase, there is a shift in the potential towards less positive values. This is also true in the case of any bare electrode, such as Au. For the MCPEI electrode, at low pH values, peak heights are low, and this can be attributed to an absence or reduced complexation of Pb^2+^ on the electrode surface while the mechanism is assumed to occur through diffusion [130].

Several voltammetric studies have been performed to concurrently determine ascorbic acid, uric acid, and dopamine. Studies of cyclic voltammetry using carbon paste electrode with formers such as 2,2′-(1,8-octanediylbisnitriloethylidine)-bis-hydroquinone and tetrabromo-p-benzoquinone have been used effectively [131,132]. The molded electrode enhances the production of current, contributing to the efficient detection of ascorbic acid in the mixture. The same sample has been studied by using cyclic voltammetry with a glassy carbon electrode molded with 0.1 mol/L H_2_SO_4_ solution in phosphate solution. The results have showed that the molded electrode interacts with H_2_SO_4_ to form a redox pair, resulting in active groups that reduce the oxidation potential when compared to the interactions of the non-molded electrode. The detection of all three compounds has been successful [133]. A simultaneous identification of all three compounds was also carried out with a molded glassy carbon electrode with LaFeO_3_ nanoparticles [134]. A combination of cyclic voltammetry and differential pulse voltammetry with a glassy carbon electrode molded with graphene/Pt nanoparticles was performed in the same sample [135]. In a bovine embryonic serum sample, the three compounds were detected by using differential pulse voltammetry with a glassy carbon electrode molded with helical carbon nanotubes. This method proved quite effective and showed great sensitivity [136]. Another study of this mixture used square-wave pulse voltammetry and molded glassy carbon electrode with Ni-poly(1,5-diaminonaphthalene) nanoparticles in NaOH 0.1 mol/L [137].

Ascorbic acid was successfully detected in a mixture with tryptophan and paracetamol by using square-wave pulse voltammetry and with a carbon paste electrode molded with multi-walled carbon nanotubes. A selective assay of ascorbic acid was carried out with this method [138]. By using square-wave pulse voltammetry, ascorbic acid was also determined in a mixture with rutin, using a carbon paste electrode molded with a carbon-chitosan nanotube film [139]. The simultaneous determination of ascorbic acid and dopamine in pharmaceutical products was conducted via the application of cyclic voltammetry with a glassy carbon electrode molded with a polymerized (poly)caffeic acid film. The polymerized film enhanced the rate of electron transfer at the electrode, resulting in the increased selectivity of the method [140]. On the other hand, the simultaneous determination of ascorbic acid and paracetamol was done by using differential pulse voltammetry with glassy carbon electrodes molded with carbon nanotubes [141].

Another study with differential pulse voltammetry was applied for the simultaneous determination of ascorbic acid and uric acid using a carbon paste electrode the surface of which was modified by adsorption of the surfactant chitosan-cetylpyridinium bromide. Both compounds were selectively identified by this method [142]. The same mixture was also studied by using linear sweeping voltammetry using a molded copper electrode with dimercaptothiadiazole, which effectively separated the peaks of the two substances [143]. In another cyclic voltammetry study to detect ascorbic acid and dopamine in pharmaceuticals and foods, the carbon paste electrode was successfully modified with a copper binuclear complex, which effectively reduced the potentials of redox reactions [144]. The mixture of ascorbic acid and dopamine was also successfully studied with differential pulse voltammetry, with an Au electrode molded with Au nanoparticles, where an effective separation of the peaks of the two substances took place [145].

In another study, a combination of differential pulse voltammetry and cyclic voltammetry was applied for the simultaneous determination of ascorbic acid, uric acid, and epinephrine. A glassy carbon electrode molded with a film of carbon nanotubes was used, thanks to which low detection limits were obtained [146]. These two techniques were also combined with a SiO_2_/Nb_2_O_5_-coated carbon electrode, which improved the electron transfer from ascorbic acid to the electrode surface. Covalent bonds may develop between ascorbic acid and Nb_2_O_5_, increasing the rate of oxidation. Nb_2_O_5_ helped to reduce the oxidation potential of ascorbic acid, which shifted to smaller values. The use of this electrode proved quite effective in determining ascorbic acid [147].

Many studies have been done to determine ascorbic acid in juices with various combinations of techniques and electrodes. Cyclic voltammetry has been applied in combination with carbon paste electrodes, while for low concentrations of ascorbic acid, modified graphite electrodes with manganese dioxide have been used in phosphate buffers (pH = 7.2) [148]. Linear sweeping voltammetry using an Au electrode has also been applied. Differential pulse voltammetry has been applied to juices and wines using glassy carbon electrode and Pt microelectrodes molded with polyvinyl sulfonium and polystyrene sulfonium film, which increased the functionality of the electrode [149,150,151]. The effective quantification of ascorbic acid has also been done in plants of the Rosa family via application of differential pulse voltammetry and square-wave pulse voltammetry with hyaline carbon electrodes [152]. In a recent study done to determine ascorbic acid in the Rosa canina plant, a square-wave pulse voltammetry was applied with a graphene oxide paste electrode molded with a film of Mn(II) complex. The method was effective, and no interference from the presence of other substances in the sample was detected. The detection limits were low (1.2888 µg/mL) and the determination of ascorbic acid was successful [153]. Another study conducted a cyclic voltammetric study of ascorbic acid using a polymelamine/gold nanoparticle modified carbon paste electrode (PM/AuNPs/CPE) in phosphate buffer solution of pH = 7.0. The electrode favored the oxidation of ascorbic acid and significantly increased the peak current, while the oxidation potential shifted to more negative potentials [154]. In a recent study, a combination of cyclic voltammetry and square-wave pulse voltammetry was applied, with a microelectrode made from pyrolytic graphite sheet (PGS), to quantify ascorbic acid in real samples. The method proved to be quite sensitive, showing low sensitivity thresholds and a linear response across a wide range of concentrations [155].

#### 3.2.2. Vitamin B1 (Thiamine)

The determination of thiamine in pharmaceuticals and juices has been studied by adsorptive stripping voltammetry at a glassy carbon electrode naked and molded with Pb^2+^ film, in acetate buffer of 0.05 mol/L and pH = 5.6. On the molded electrode a reduction peak with a potential of −1.25 V versus the Ag/AgCl electrode appeared, while on the bare electrode no peak was observed. This showed the effectiveness of Pb^2+^ film as a formator. One possible mechanism indicates that thiamine is likely adsorbed to the Pb^2+^ film but not to the electrode. At Pb^2+^ concentration of between 1 × 10^4^–1.5 × 10^4^ mol/L, the maximum reduction of thiamine was observed [156].

In another study, the electrochemical behavior of thiamine was studied by using square-wave voltammetry at a Cys/SAM shaped Au electrode (Cys/SAM/Au) in an alkaline environment (pH = 11.0). The results showed that the Cys/SAM/Au molded electrode had electrocatalytic activity with versus thiamine and thatthe process was controlled by adsorption. With an increase in pH (from 7.4 to 11.9) the anodic peak shifted to more negative values. This method was a simple, fast, and selective high-precision technique for determining thiamine in the presence of other vitamins in pharmaceutical formulations, and could be applied in clinical analysis and drug analysis [157].

The successful determination of vitamin B1 in a mixture of vitamins B2 and C was also achieved by using adsorptive stripping voltammetry with a glassy carbon electrode molded with solid silver amalgam film (AgLAF-AgSAE), which caused very good separation of the peaks of vitamin B1 and C and showed satisfying detection limits [44]. The use of DNA/MWCNT-molded carbon paste electrode also appeared effective [158].

#### 3.2.3. Vitamin B2 (Riboflavin)

The determination of riboflavin in pharmaceutical formulations has been done by using cyclic voltammetry using an optical sensor made of cyclodextrin wherein riboflavin is encapsulated in cyclodextrin [159]. The determination of riboflavin in real samples has been mainly done by adsorptive stripping voltammetry with an Hg electrode at basic conditions (a bare glassy carbon electrode has also been used), in H_2_SO_4_ buffer of 0.5 M. The mechanism has showed the reversible reduction of two electrons and two protons in the solution while a small amount of riboflavin has been adsorbed to the bare electrode [160]. This electrode has also been used to determine riboflavin in breast milk [161]. Riboflavin has also been studied with a pretreated glassy carbon electrode (PGCE). In one such study, the described mechanism showed a reversible reduction of riboflavin to quinone on the carbon surface with a potential of +0.4 V. Riboflavin and its reduction product adsorbed much more readily to the activated electrode than to the untreated electrode [159]. The mechanism of adsorption of vitamin B2 on the hanging Hg drop electrode in an aqueous mean is due to the redox reaction of 2e^−^/2H^+^ of the flavin portion, accompanied by strong adsorption on the electrode surface of both the redox form of quinone and the hydroquinone of vitamin B2. In addition to conventional square-wave pulse voltammetry, the mechanism of electrodes has been investigated in more detail with cyclic square-wave voltammetry (CSWV) [162]. Voltammetric parameters are affected by vitamin B2 concentration, pH, and accumulation time. With an increase in the concentration of the adsorbed materials on the electrode surface, the peaks shift to less negative potentials. This demonstrates that interactions between the adsorbed species take place. With an increase in pH the transfer of electrons becomes faster, and the adsorption of riboflavin becomes stronger, than at a lower pH. The increase in accumulation time forms a solid film on the surface of the electrode, allowing it to require higher energy for the redox reaction, thus shifting the potential towards negative values. However, by increasing the surface concentration, repulsive interactions between adsorbed molecules become important [162].

The determination of riboflavin has also been studied by using adsorptive voltammetry with hyaline carbon electrode molded with nanomaterials which enhance the catalytic action of riboflavin. Such nanomaterials include polythiophene nanotubes, metal nanocrystals, oleylamine nanoparticles (OLA), Cr/SnO nanoparticles, NiO/MWCNTs, and poly(3,4-ethylenedioxythiophene)/zirconia (PEDOT/ZrO_2_NPs) [163]. Additionally, a study was conducted using differential pulse voltammetry with carbon paste electrodes molded with Co-Y zeolites. The Co-Y zeolite interacted with riboflavin, resulting in increased electrode selectivity and sensitivity [94]. Recently, a sensor based on the transition nanocomposite materials made of metal binary oxide (ZnO-MnO) was synthesized with a CSNs shell core on the surface of a glassy carbon electrode. This sensor was used to determine riboflavin with cyclic voltammetry, differential pulse voltammetry, and linear sweeping voltammetry with excellent stability, repeatability, selectivity, and sensitivity [91].

#### 3.2.4. Vitamin B3 (Nicotinic Acid/Niacin)

The determination of vitamin B3 in biological samples, food, and pharmaceutical tablets has been an object of much interest. The use of electrochemical sensors on a carbon paste electrode is a very good method of determining vitamin B3 in pharmaceutical products due to their simplicity, selectivity, high sensitivity, and low cost. A study that used cyclic voltammetry to determine vitamin B3 in syrup samples used CoTMPP-molded graphite paste microelectrode (CoTMPP/Nafion/GPE), which enhanced the electrocatalytic response of the vitamin’s reduction current. The corresponding cyclic voltammograms were recorded in 0.1 mol/L NaNO_3_ solution, pH = 3.9, using GPE, CoTMPP/GPE, and CoTMPP/Nafion/GPE at a scan rate of 0.1 V/s. The utilized method proved too sensitive and simple for the selective determination of nicotinic acid [164]. The determination of nicotinic acid in pharmaceutical products was also done using an Au electrode and the application of cyclic voltammetry, while in food, an Au electrode molded with thioglycolic acid and an adsorptive stripping voltammetry was applied [165,166].

#### 3.2.5. Vitamin B5 (Pantothenic Acid)

The detection of D-Panthenol in a urine sample was done by using square-wave pulse voltammetry with a glassy carbon electrode at pH = 4.2. The method proved to be quite sensitive and with low limits of detection (5.0 × 10^−7^ M) [91].

#### 3.2.6. Vitamin B6 (Pyridoxine)

The determination of vitamin B6 in pharmaceutical products has been carried out with applications of cyclic voltammetry and differential pulse voltammetry using molded glassy carbon electrodes with materials such as vanady(IV)-salen complex, carbon nanotube, Ru(bpy)_3_^3+^ with diamond-boron complex, Prussian blue, poly-methylene blue, and graphite-modified carbon paste electrode. A study conducted with differential pulse voltammetry for the simultaneous determination of vitamins B6 and C used a carbon paste electrode molded with ZnO/Cuo nanocomposites with 2-[ferrocenylethynyl]fluorine-9-one, ZnO/Cuo in an ionic liquid, 2FE/ZC/IL/CPE. The results showed good sensitivity and competent separation of the peaks [93]. On the other hand, in other differential pulse voltammetric studies, Au electrodes fused with carbon nanotubes and Au-CuO molded carbon nanotubes have also been effectively used. In foods (energy drinks and cereals) and dietary supplements (multivitamins), B6 has been determined by using differential pulse voltammetry with printed disposable silk electrodes. This method proved to be quite effective, selective, repeatable, and with low sensitivity limits (1.5 × 10^−6^ M) [167].

#### 3.2.7. Vitamin B7 (Biotin)

In food, the electroanalytical study of biotin has been done by using differential pulse voltammetry with a film biosensor that was synthesized from ionic liquid-chitin and modified with Pd-Fe-Ni nanoparticles [168]. In another study, the determination of biotin in blood plasma was performed using a boron-doped diamond electrode (BDD) on the surface of which a Nafion layer had formed. The detection limit of the method was quite low (up to 5 nM), and it has been quite an effective method in clinical analyses [169].

#### 3.2.8. Vitamin B9 (Folic Acid)

The detection of vitamin B9 in pharmaceuticals and foods has been done primarily with square-wave voltammetry, differential pulse voltammetry, and cyclic voltammetry, but also with a combination of these. In each case, different working electrodes are used. In studies with square-wave pulse voltammetry, carbon paste electrodes molded with Pt:Co nanomaterials have been used [170]. In studies that have combined square-wave pulse voltammetry and cyclic voltammetry, carbon paste electrodes molded with complexed carbon nanotubes with Ru(II)ZnO have been used [171]. In studies combining square-wave and differential pulse voltammetry, carbon paste electrodes modified with nanomaterials have been used [172,173]. In differential pulse voltammetry studies, carbon paste electrodes molded with polymer films, TiO_2_ nanomaterials, and magnetite nanoparticles have been used, and Au electrodes molded with nanomaterials have also been used [174,175,176,177]. In studies combining differential pulse voltammetry and cyclic voltammetry, carbon paste electrodes molded with carbon nanotubes have been used [178]. Additionally, the determination of folic acid in food has also been studied by using adsorptive voltammetry with film-Bi-molded Au electrodes, Ag amalgams, or Hg [169,179,180]. In a recent study involving pharmaceutical tablets, the detection of vitamin B9 was done using a combination of cyclic voltammetry and differential pulse voltammetry in a phosphate buffer (pH = 7), using as a sensor an electrochemically polymerized tyrosine film on a graphite substrate. This method turned out to be quite sensitive and repeatable [181].

Additionally, the simultaneous determination of folic acid and riboflavin has been done by using cyclic voltammetry using Hg electrodes [182]. The results have showed that both vitamins are strongly adsorbed on the surface of the electrodes, and that this is due to the presence of the aromatic rings. Adsorbed molecules can then be measured by using the technique of adsorptive stripping voltammetry, which has been found to be quite effective for the simultaneous determination of folic acid and riboflavin, in the presence of excess electroactive substances in solution with great precision and selectivity. In another study, the successful determination of folic acid in a mixture with ascorbic acid and riboflavin was performed by using adsorptive stripping voltammetry involving glassy carbon electrodes molded with Pb^2+^ [183].

#### 3.2.9. Vitamin B12 (Cobalamin)

The determination of vitamin B12 in blood plasma and urine has been studied by using differential pulse voltammetry with an electrochemical sensor, molded with polypyrrole, which is prepared with carbon fiber paper, and then by applying the electro deposition of palladium-gold nanoparticles (PdAu). A study on this has showed good selectivity in the presence of other substances [184]. Similarly, vitamin B12 has been detected in foods with ferromagnetic nanoparticles from triazine dendrimers (FMNPs@TD) using a combination of cyclic voltammetry and differential pulse voltammetry techniques [109]. The selective detection of B12 in injectable drugs has also been performed by using cyclic voltammetry with glassy carbon electrodes, molded with fluorescent nanosensors without labeled carbon. The fluorescence intensity of CD charts decreases as the concentration of vitamin B12 increases, showing a linear relationship in the range from 0 to 60 M [185]. In another recent study, the electrochemical determination of cyanocobalamin with square-wave pulse voltammetry was applied using an electrode of a carbon paste molded with a Mg complex film with thiophene-2-carboxylic acid and triethanolamine substituents in KCl. The method proved to be selective, as the resulting interference did not affect the detection of cyanocobalamin, had low detection limits and low cost, and was effectively applied to pharmaceutical tablets and dietary supplements [186]. In another study, the determination of cyanocobalamin was performed in human urine samples by using a combination of cyclic voltammetry and adsorptive transfer square wave voltammetry, using a DNA electrochemical biosensor and a modified carbon paste electrode. The electrode was modified with an electrochemically produced polymer (Mn(thiophenyl-2-carboxylic acid)_2_(triethylonamine)), while cyanocobalamin was immobilized onto the modified electrode. The method proved to be simple, fast, selective, and with low detection limits (1210 µg/L) [187].

The determination of B12 in pharmaceuticals has been done by using cyclic voltammetry, with an Au electrode modified with mercaptoacetic acid at 0.01 mol/L HCl. The corresponding voltammogram has showed three reduction peaks with potentials of 0.21 V, 0.16 V, and −0.41 V, with a total exchange of two electrons. The main form of Co^3+^ is directly reduced to 0.21 V via transfer of an electron by CN^−^ cleavage into the cyanocob(II)alamin. The detection limit of this method is quite low (1.0 × 10^−9^ mol/L) [188]. In previous studies on the determination of vitamin B12 in pharmaceuticals, anodic adsorptive voltammetry was applied with disposable graphite screen-printed electrodes that had been modified with Bismuth film [189]. Square-wave voltammetry using a disposable pencil graphite electrode modified with peptide nanotubes was also effective [190]. Additionally, a successful combination of cyclic voltammetry and square-wave voltammetry with a disposable pencil graphite electrode modified with carbon-chitosan nanotubes has been applied [191]. In a study of dietary supplements, the determination of vitamin B12 was done through the application of cyclic voltammetry with a boron-doped diamond electrode wherein the determination of the redox pair Co^+^→Co^2+^ was monitored. The corresponding voltammogram obtained at pH = 5.0 showed two oxidation peaks for the pairs Co^+^→Co^2+^ and Co^2+^→Co^3+^ with potentials −0.74 V and +0.18 V, respectively, and two reduction peaks for the pairs Co^3+^→Co^2+^ και Co^2+^→Co^+^ with potentials −0.12 V and −0.75 V, respectively [192]. All relevant analytical figures of merit are being summarized in Table 2.

## 4. Conclusions

In this study, a bibliographic review of the electrochemical techniques that have been applied for the determination of vitamins (fat-soluble and water-soluble), mainly focusing on voltammetric techniques, was carried out. Voltammetric techniques are fast, have low cost, do not destroy the sample in most cases, are repeatable, and have great sensitivity and selectivity. The electrochemical properties of vitamins favor their study and determination by using voltammetry, and the most common techniques that have been used on them are cyclic voltammetry (CV), differential pulse voltammetry (DPV), square-wave voltammetry (SWV), and anodic stripping voltammetry (ASV). The most commonly used electrodes are glassy carbon material and carbon paste, bare and molded. Pt electrodes have also been used effectively in some determinations. The use of nanoparticles and carbon nanotubes as modifiers has proved to be more effective for the selective determination of a studied vitamin than for a mixture of vitamins, even with low sensitivity limits. The mechanisms carried out at the working electrode surface differ depending on the vitamin being studied. They can include a simple electron transfer (as in B2, B3, B7, C, and D), a simultaneous oxidation and reduction (as in B1, B5, B6, B9, and E), or two successive reductions (as in K and B12), while vitamin A has complex electrochemical properties. The use of electrochemical analysis methods is an attractive alternative to conventional analytical techniques due to the much faster speed and lower cost of the former. The simultaneous identification of many vitamins is a challenge, but the results of recent studies are promising. Many new electrode materials, especially those that are nanostructured, have been introduced that have increased selectivity and sensitivity, also allowing the quantification of vitamins in true samples for concentrations much lower than the other components.

The range of applications is particularly wide, going from pharmaceutical and nutritional supplements to more complex samples such as food and biological fluids. However, most of the proposed methods still require pretreatment and/or the dilution of the true samples for analysis. The challenge for future studies will be the development of sensors capable of identifying vitamins directly in real samples, increasing the speed of analysis, the development of portable devices in combination with the use of one-shot sensors, all of which will open new perspectives. Electrochemical techniques have proven to be excellent candidates for such applications, as small portable potentiostats are already available in the market. The application of electrochemical techniques in the determination of vitamins in pharmaceutical formulations, foods, and dietary supplements, even in biological samples, is very important, because in these samples, the vitamins exist even in trace amounts. Since the listed methods used show selectivity at low detection limits, the quantification of vitamins can also be accomplished by using them. These conclusions allow electrochemical techniques to represent important assets in vitamin analysis.

## Figures and Tables

**Table 1:** The vitamins, their dietary sources, and their biological functions or reactions.

**Fat-Soluble Vitamins**			
**Groups**	**Vitamers**	**Dietary Sources**	**Biological Functions or** **Reactions**
Vitamin A	Retinol Retinal Retinoic acid Provitamins: (β-carotene, cryptoxanthin)	Green, yellow, orange plant tissues (fruits and vegetables; carotenoids), milk, red meat, eggs, poultry meat, fatty fish and shellfish, margarine, breast milk, production from intestine-adapted mutant strain *E. Coli* [1]	Regulation of epithelial cell differentiation Photosensitive retinal pigment
Vitamin D	Cholecalciferol (D3) Ergocalciferol (D2)	Ergocalciferol: plants, fungi, molds, lichens, brates (snails, worms), mushrooms. Cholecalciferol: animal tissues, fish liver and oils, fatty fishes, eggs, chicken, beef, pork, plants of the family Solaneceae, enriched food oils [1]	Calcium homeostasis Bone metabolism (calcium mobilization) Transcription factor
Vitamin Ε	α-Tocopherol β-Tocopherol γ-Tocopherol	Green plants, plant oils, wheat germ oil, rice bran oil, sunflower oil, corn oil, mayonnaise, almonds, milk and milk products [1]	Antioxidant protector for membranes
Vitamin Κ	Phylloquinones (Κ1) Menaquinones (Κ2) Menadione (Κ3)	Green leafy vegetables (e.g., spinach, kale, broccoli, Brussels sprouts), vegetable oils, margarine, bacterially fermented foods (e.g., cheese, sauerkraut), poultry and pork products [1]	Blood clotting Calcium metabolism
**Water-Soluble Vitamins**			
Vitamin C	Ascorbic acid Dehydroascorbic acid	Fruits, vegetables, organ meats (liver, kidney), fresh tea leaves, berries, citrus [1]	Reductant in hydroxylations in the formation of collagen and carnitine and in the metabolism of drugs and steroids
Vitamin Β_1_	Thiamin	Yeasts, liver (especially pork liver), cereal grains, pork, cured ham [1]	Coenzyme for oxidative decarboxylation of 2-keto acids (e.g., pyruvate, 2-keto-glutarate) and for pyruvate decarboxylase and transketolase
Vitamin Β_2_	Riboflavin	Green leafy vegetables (broccoli, cabbage, carrots, spinach], fruits, meat, eggs, dairy products (milk, yogurt, cheese), cereals (wheat, rice, oats) [1]	Coenzyme in redox reactions of fatty acids and the tricarboxylic acid (TCA) cycle
Vitamin Β_3_	Niacin Nicotinic acid Nicotinamide	Brewer’s yeast, beans, spinach, tomato, fruit, nuts, eggs, mushrooms, meat, milk [1]	Coenzyme for several dehydrogenases, significant role in the oxygenation of tissues, participates in the metabolism of proteins, fatty acids, and carbohydrates. A component of hydrogen-carrying coenzymes (NAD, NADP) involved in glycolysis, the Krebs cycle, the synthesis of fatty acids
Vitamin B_5_	Pantothenic acid	Meat (liver, heart), mushrooms, avocado, eggs, nuts, broccoli, yeast, rice, peanut flour, molasses, concentrated soluble fish [1]	Coenzyme in fatty acid
Vitamin B_6_	Pyridoxine Pyridoxal Pyridoxamine	Meats, whole grain products (wheat), vegetables (asparagus, beans, cabbage), nuts, cereals, fish [1]	Coenzyme in amino acid
Vitamin Β_7_	Biotin	Royal jelly, brewer’s yeast, milk, liver, egg yolk, vegetables (broccoli, spinach asparagus, carrots, potatoes), fruit, beans, milk, cheese [1]	Coenzyme for carboxylations (e.g., acetyl CoA/malonyl CoA conversion)
Vitamin B_9_	Folate Pteroylglutamic acid Pteroylpolyglutamates	Liver, mushrooms, green leafy vegetables, soybean meal, milk and milk products, yeast, beans, fruits [1]	Coenzyme in single-carbon
Vitamin B_12_	Cyanocobalamin	Fermented foods, liver, dairy products, meat, egg, fish, shellfish, edible algae, mushrooms, fermented soy products, fruits, tea leaves, spinach, broccoli, asparagus, cyanobacteria (spirulina) [1]	Coenzyme in the metabolism of propionate, amino acids, and single-carbon units

**Table 2:** Aggregated results from the voltammetric studies of vitamins (fat/water-soluble) in samples.

Vitamin	Voltammetric Technique [ab.]	Working Electrode	Sample	Reference
Vitamin A	CV	Glassy carbon	Retinol/Vitamin Ε	[60]
	SWV	Carbon paste modified with Pt: Co nanoalloy	Foods	[101]
	DPV	Carbon paste modified with surfactant sodium dodecyl sulfate [CPE/SDS]	-	[102]
Vitamin D	SWV	Boron Doped Diamond Electrode	-	[62]
	CV	Glassy carbon	-	[55,61,66,67]
	CV, DPV	Glassy carbon modified thin synthetic indium-titanium oxide	D2	[103]
	DPV	Glassy carbon modified with PANnFs and NPsFe_3_O_4_	D3	[104]
	DPV	Glassy carbon ΙTO, modified with Asp-Gd_2_O_3_NRs sensor	D3	[61]
	CV, DPV	Glassy carbon modified with Ni[OH]_2_ particles in SiO_2_/ graphene oxide organic-inorganic matrix	Pharmaceutical	[105]
	CV	Glassy carbon modified with nanosensor employing fullerene-C60 and bimetallic nanoparticles composite film	Blood	[106]
	AdSV	Screen-printed carbon coated with MIP and p-phenylenediamine-resorcinol	Real samples	[107]
	CV, DPV	nCeO_2_/CC immunosensor	Biological samples	[108]
Vitamin Ε	DPV	Planar Pt	Vegetable oils and fats	[55]
	CV, DPV	Au modified with Pan/c-Al_2_O_3_	Food supplements	[109]
	SWAdSV	Glassy carbon	Vitamins Ε και Κ	[110]
	SWV	CF disk UME in Bz/EtOH [1:2]	α-, β-, γ-, δ-tocopherols in oil samples	[111]
	SWAdSV	Glassy carbon	Vitamins Ε and Κ in food supplements	[115]
Vitamin Κ	CV	Glassy carbon	-	[115]
	SWAdSV	Glassy carbon	Plant based foods	[116]
	CV	Glassy carbon	Pharmaceutical Products and Foods	[115,117]
	CV	Pencil graphite modified with silver nanoparticles and 2-amino-5-chloro benzophenone	Blood plasma	[118]
	CV, LSV	Glassy carbon modified with PEDOT	Poultry drugs	[119]
	SWAdSV	Glassy carbon	Vitamins A, D, E, K	[120]
Vitamin C	SWV	Carbon paste modified with Fe[III]-Y	-	[88]
	CV	Carbon paste modified with p-tert-butylcalix [4] arene	Commercial Samples	[130]
	CV, DPV	Carbon paste coated with NPsZnO-Pd	Fruit Juices, creams	[121]
	CV, DPV	Printed carbon electrode bare and modified with ZnO/Al_2_O_3_	Real samples	[83]
	DPV	Carbon paste modified with graphene	Pharmaceutical Products	[122]
	DPV	Carbon paste modified with porphyrins	Pharmaceutical Products	[123]
	CV	Glassy carbon modified with poly[bromocresol purple] film	-	[124]
	CV	Glassy carbon modified with poly [3-[5-chloro-2-hydroxyphenylazo]-4, 5-dihydroxynaphthalene-2, 7-disulfonic acid] film	Ascorbic acid in a mixture of substances	[125]
	CV	Pt coated with iodine	MultiVitamins [B1, B6, B9, B12, C]	[128]
	CV, DPV	Carbon paste modified with multi-wall carbon nanotubes and graphite	Pharmaceutical formulations και Foods	[129]
	CV	Carbon paste modified with 2,2′-[1,8-octanediylbisnitriloethylidine]-bis-hydroquinone and tetrabromo-p-benzoquinone	Mixture of ascorbic acid, uric acid and dopamine	[131,132]
	CV	Glassy carbon modified with H_2_SO_4_	-	[133]
	CV	Glassy carbon modified with LaFeO_3_ nanoparticles	-	[134]
	CV, DPV	Glassy carbon modified with graphene/Pt nanoparticles	-	[135]
	SWV	Glassy carbon modified with Ni-poly [1,5-diaminonapthalene] nanoparticles	-	[137]
	DPV	Glassy carbon modified with helical carbon nanotubes	Ascorbic acid, uric acid and dopamine in a bovine foetal serum sample	[136]
	SWV	Carbon paste modified with multi-wall carbon nanotube	Ascorbic acid, tryptophan, paracetamol	[138]
	SWV	Carbon paste modified with multi-walled carbon nanotubes–chitosan composite film	Ascorbic acid-Rutin	[139]
	CV	Glassy carbon modified with poly [caffeic acid]	Ascorbic acid and dopamine in Pharmaceutical Products	[140]
	DPV	Glassy carbon modified with carbon nanotubes	Ascorbic acid, paracetamol	[141]
	DPV	Carbon paste modified with cetosan-cetylpyridinium bromide	Ascorbic acid and uric acid	[142]
	LSV	Au modified with dimercaptothiadiazole	-	[143]
	CV	Carbon paste modified with novel bicopper complex	Ascorbic acid and dpamine in Pharmaceutical Products και Foods	[144]
	DPV	Au modified with self-assembled Au nanoparticles	Ascorbic acid abd dopamine	[145]
	DPV, CV	Glassy carbon modified with multiwall carbon nanotubes with nafion	Ascorbic acid, uric acid, epinephrine	[146]
	DPV, CV	Carbon coated with SiO_2_/Nb_2_O_5_	-	[147]
	CV	Carbon paste	Juices	[148]
	CV	Graphite modified with Manganese dioxide	Juices	[148]
	LSV	Gold	Juices	[148]
	DPV	Glassy carbon	Juices and wines	[144,145,146,147,148,149,150,151]
	DPV	Pt microelectrodes modified with polyvinyl sulfonium and polystyrene sulfonium film	Juices and wines	[149,150,151]
	DPV, SWV	Glassy carbon	Plants of the *Rosa* family	[152]
	SWV	Graphene oxide paste modified with manganese[II] complex	Rosa canina	[153]
	CV	Carbon paste modified with PM/AuNPs	-	[154]
	CV, SWV	Microelectrode made from pyrolytic graphite sheet [PGS]	Real samples	[155]
	DPV	Glassy carbon modified with MXene powder (titanium carbide)	Human urine samples	[193]
Vitamin Β_1_	AdSV	Carbon paste	-	[89]
	CV	Carbon paste modified with MnPC	-	[89]
	CV	Platinum	Thiamine Pyrophosphate	[48]
	AdSV	Glassy carbon modified with Pb^2+^ film	Pharmaceutical Products and Juices	[156]
	SWV	Au modified with Cys/SAM	Pharmaceutical formulations	[157]
	AdSV	Glassy carbon modified with AgLAF-AgSAE	Mixture vitamins B1, B2, C	[44]
	AdSV	Carbon paste modified with DNA/MWCNT	-	[158]
Vitamin Β_2_	CV	Optical sensor made of cyclodextrin	Pharmaceutical formulations	[159]
	AdSv	Hg/Bare glassy carbon	Real samples	[160]
	AdSV	Bare Glassy carbon	Breast milk	[161]
	ASV	Glassy carbon modified with nanomaterials	-	[162]
	DPV	Carbon paste modified with Co zeolites	-	[94]
	CV, DPV, LSV	Glassy carbon modified with ZnO-MnO/CSNs	-	[91]
Vitamin Β_3_	DPV	Glassy carbon	-	[55]
	CV	Hg/Dropping Hg/Pt	-	[55]
	CV	Microelectrode graphite paste modified with CoTMPP/Nafion	Syrup	[164]
	CV	Gold	Pharmaceutical Products	[165,166]
	ASV	Au modified with thioglycolic acid	Foods	[165,166]
Vitamin Β_5_	CV, DPV, LSV	Carbon paste modified with cobalt[II]oxide catalyst	D-panthenol	[55]
	SWV	Glassy carbon	Urine	[91]
Vitamin Β_6_	SWV	Carbon paste modified with ZnO/Cuo	Mixture of vitamins C and Β6	[93]
	DPV	Au modified with carbon nanotubes	-	[93]
	DPV	Glassy carbon modified with dsDNA	-	[93]
	DPV	Disposable printed silk	Foods (Energy drinks, cereals), multivitamins	[167]
Vitamin Β_7_	SWV	Film biosensor modified with Pd-Fe-Ni NPs	Foods	[168]
	CV, DPV	Diamond with mixture of boron/Nafion	Blood plasma	[169]
Vitamin Β_9_	CV	Au modified with MBT/SAM film	-	[55]
	CV	Dropping Hg	-	[55]
	CV	Au modified. with multi-wall carbon nanotube Au/NPs,	-	[97]
	CV, AdSV	Hg electrode	Folic acid, riboflavin	[182]
	ASV	Glassy carbon modified. with Pb^2+^ film	Mixture of ascorbic acid and riboflavin	[183]
	SWV	Carbon paste modified. with Pt:Co nanomaterials	Pharmaceutical Products, Foods	[170]
	SWV, CV	Carbon paste modified. with Ru[II]ZnO complex carbon nanotubes	Pharmaceutical Products, Foods	[171]
	SWV, DPV	Carbon paste modified. with nanomaterials	Pharmaceutical Products, Foods	[172,173]
	DPV	Carbon paste modified. with polymeric film, TiO_2_ nanomaterials, magnetite nanoparticles/Au modified with nanomaterials	Pharmaceutical Products, Foods	[174,175,176,177]
	DPV, CV	Carbon paste modified with carbon nanotubes	Pharmaceutical Products, Foods	[178]
	ASV	Au modified. with Bi-film, Ag or Hg amalgams	Foods	[177,179,180]
	CV, DPV	Polymerized tyrosine film on graphite substrate	Pharmaceutical tablets	[181]
Vitamin Β_12_	DPV	Electrochemical sensor modified. with polypyrrole and PdAu NPs	Blood plasma, Urine	[184]
	CV, DPV	Ferromagnetic nanoparticles from triazine dendrimers [FMNPs@TD]	Foods	[109]
	CV	Glassy carbon	Injectable Drugs	[185]
	SWV	Carbon paste modified. with Mg complex film with thiophene-2-carboxylic acid and triethanolamine substituents	Pharmaceutical tablets, Food supplements	[186]
	AdSV	Carbon paste modified. with [Mn [thiophenyl-2-carboxylic acid]-2 [triethylonamine] polymer and DNA biosensor	Urine samples	[187]
	CV	Au modified. with mercaptoacetic acid	Pharmaceutical Products	[188]
	ASV	Disposable carbon mesh modified with bismuth film	Pharmaceutical Products	[189]
	SWV	Graphite modified with peptide nanotubes	Pharmaceutical Products	[190]
	SWV	Pencil Graphite modified with carbon nanotube-chitosan	Pharmaceutical Products	[191]
	CV	Diamond with boron admixture	Food supplements	[192]

CV; cyclic voltammetry, SWV; square wave voltammetry, ASV anodic stripping voltammetry, AdSV adsorptive voltammetry, DPV; Differential pulse voltammetry.

## Data Availability

Not applicable.
